# Isothermal close space sublimation for II-VI semiconductor filling of porous matrices

**DOI:** 10.1186/1556-276X-7-409

**Published:** 2012-07-23

**Authors:** Vicente Torres-Costa, Claudia de Melo, Aurelio Climent-Font, Fernando Argulló-Rueda, Osvaldo de Melo

**Affiliations:** 1 Applied Physics Department, Faculty of Sciences, Universidad Autónoma de Madrid, Cantoblanco, Madrid, 28049, Spain; 2 Physics Faculty, University of Havana, Havana, 10 400, Cuba; 3 Centro de Microanálisis de Materiales, Cantoblanco, Madrid, 28049, Spain; 4 Instituto de Ciencia de Materiales de Madrid (ICMM-CSIC), Cantoblanco, Madrid, 28049, Spain

**Keywords:** Porous silicon, II-VI semiconductors, Thin films, Nanostructures, Rutherford backscattering spectroscopy, 82.80.Yc, 81.05.Rm, 81.15.Kk

## Abstract

Isothermal close space sublimation, a simple and low-cost physical vapour transport technique, was used to infiltrate ZnTe and CdSe semiconductors in porous silicon. The structure of the embedded materials was determined by X-ray diffraction analysis while Rutherford backscattering spectra allowed determining the composition profiles of the samples. In both cases, a constant composition of the II-VI semiconductors throughout the porous layer down to the substrate was found. Resonance Raman scattering of the ZnTe samples indicates that this semiconductor grows in nanostructured form inside the pores. Results presented in this paper suggest that isothermal close space sublimation is a promising technique for the conformal growth of II-VI semiconductors in porous silicon.

## Background

Porous silicon (PS) has received a lot of interest in recent years due to its many peculiar properties. In particular, its porosity may reach up to 90% under appropriate conditions [1], and its internal surface may be as high [2] as 800 m^2^/cm^3^; this makes PS a promising material for catalytic applications and also as a matrix material for embedding different substances such as SnO_2_[3], C_60_ molecules [4] or even nematic liquid crystals [5]. The interest in embedding materials in PS originates from different reasons. For example, luminescence stability of porous silicon is related to the passivation of its internal surfaces. Also, the small pores of silicon can be used as templates for nanocrystal formation. On the other hand, potential PS-based optoelectronic devices require electrical contacts not only in the external surface on the PS film, but also in the internal walls of the pores.

The filling of intricate and large aspect ratio pores as is the case of PS is a difficult task. This is because using most traditional vapour phase deposition techniques, the vapour transport is strongly favoured near the entrance of the pores; then they eventually become obstructed, and the inner part of the pores remain empty. The use of isothermal close space sublimation (ICSS) technique for growing epitaxial or polycrystalline films in dependence of the type of substrate has been verified in previous publications [6,7]. This technique uses alternate exposure to the different elemental sources in a regime in which there is no temperature difference between the source and the substrate. This allows regulation of the growth process. For this reason we explore the possibility of filling PS layers using this technique. We presented the growth of CdSe and ZnTe semiconductors inside the pores of porous silicon using alternated evaporation of elemental Cd, Zn, Te and Se.

## Methods

Porous silicon was prepared by electrochemical etching of monocrystalline p^+^ (100) silicon wafers, in a 1:1 ethanol and HF (48 wt.%) electrolyte. The process took place in a Teflon cell under illumination provided by a 150-W halogen lamp to increase final porosity. Anodisation current was provided by a computer-controlled EG&G 263 galvanostat/potentiostat (Princeton Applied Research, Oak Ridge, TN, USA). Applied current density was 10 mA/cm^2^ for low porosity layers and 150 mA/cm^2^ for high porosity ones. This setup is known to produce homogeneous, spongelike porous silicon samples [8].

**Figure 1:**
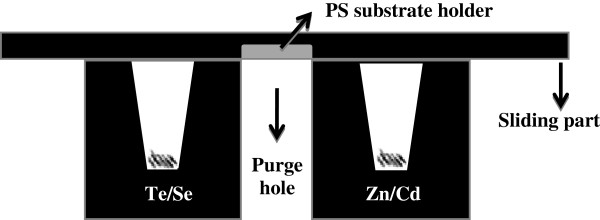
Sketch of the graphite boat used for the infiltration of the PS layers.

CdSe and ZnTe semiconductors were grown onto PS surface by ICSS technique. General details of this growth setup and process can be found elsewhere [6,7]. A graphite crucible, schematically shown in Figure 1, was placed in a plate-shaped temperature profile at 390°C for ZnTe growth and 290°C for CdSe growth. PS samples (1 × 1 cm^2^) were alternately exposed to Zn (99.99%) and Te (99.999%) and to Cd (99.99%) and Se (99.999%) elemental sources (provided by Goodfellows Cambridge Ltd., Huntingdon, England) by cyclically moving the sliding part of the boat back and forth. In between expositions to the sources, a vapour purge step was allowed, locating the substrate in an intermediate purge hole in the graphite boat. The process was performed under an Ar gas flow of about 4 cm^3^/s at near atmospheric pressure. A programmed linear actuator LA12-PLC from LINAK (Nordborg, Denmark) was used to perform the deposition cycles. Previous to the growth experiments, the PS substrates were degreased in acetone and alcohol and in some cases dipped for 15 s in HF. Exposure time to the elements and purge time were varied. The temperatures were selected taking into account previous growth experiments of thin films; they represent the lower limits of the temperature range in which CdSe and ZnTe can be grown by ICSS. The distance between sources and substrates was around 5 mm, and the growth surface was a circle of 7 mm of diameter.

Rutherford backscattering spectroscopy (RBS) analyses for the characterization of the samples were performed with a 3,035-keV α-particle beam provided by the Cockcroft-Walton tandem accelerator at the Centre for Micro-Analysis of Materials at Universidad Autónoma de Madrid. A main Si detector was placed at 170.5° scattering angle position and a second one with a variable scattering angle position was placed at 165°, giving information of the depth profile of the sample. For the analysis, RBS spectra were simulated using the SIMNRA code (Max-Plank-Institut für Plasmaphysik, Garching, Germany) [9] in order to determine the composition profiles. High-resolution scanning electron microscopy (SEM) images of the samples were obtained using a JEOL Microscope mod. JSM 6335 F (JEOL Ltd., Akishima, Tokyo, Japan). Grazing incidence X-ray diffraction (XRD) scans were taken using a Siemens D-5000 powder diffractometer (Siemens AG, Munich, Germany). Raman spectra were measured with a Renishaw Ramascope 2000 microspectrometer (Renishaw, Wotton-under-Edge, UK) and a × 100 microscope objective.

## Results and discussion

### CdSe

**Figure 2:**
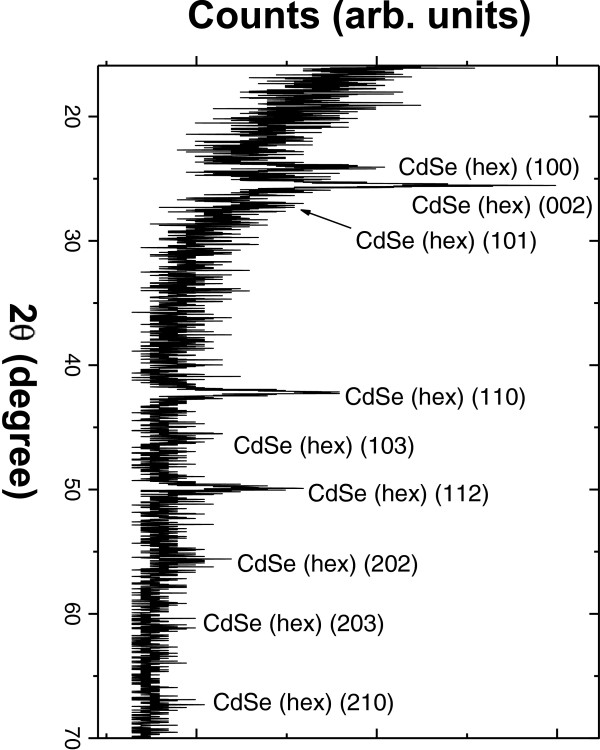
Grazing incidence XRD for a CdSe-embedded PS sample showing the wurtzite crystalline structure of CdSe.

**Figure 3:**
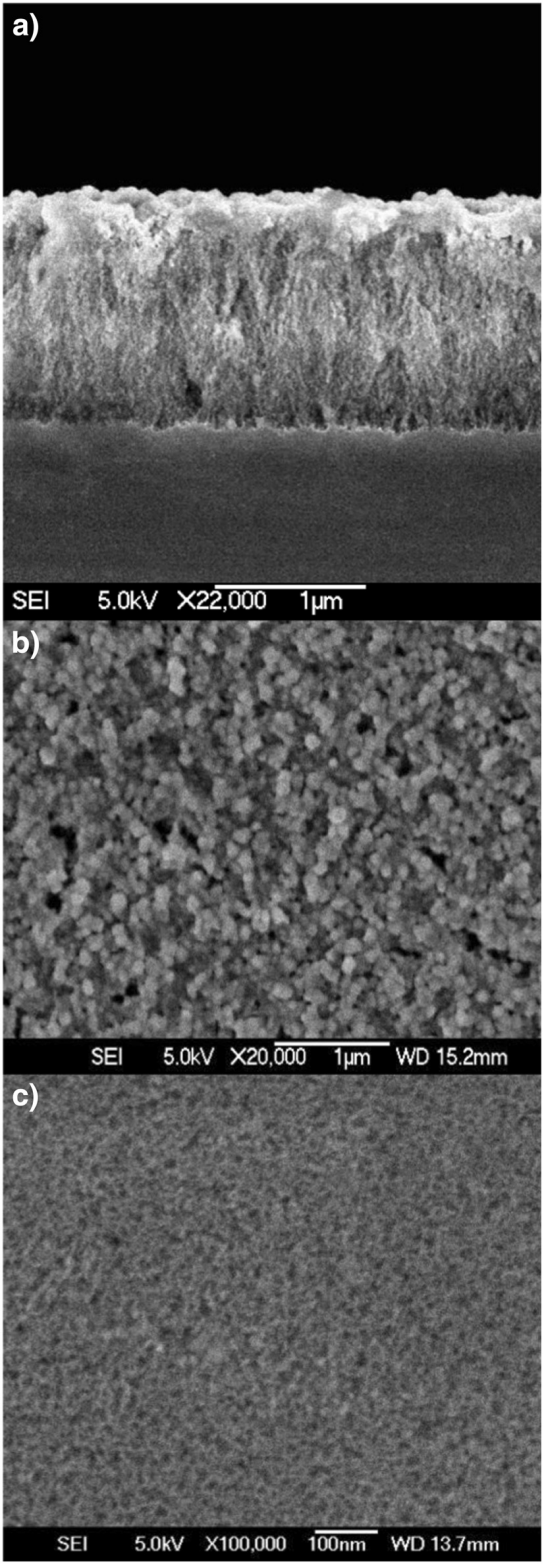
**SEM micrograph for a CdSe embedded PS layer and PS pristine layer.** Cross-section (**a**), top-view (**b**) SEM micrograph for a CdSe-embedded PS layer. (**c**) The surface of a PS pristine layer is shown for comparison.

**Figure 4:**
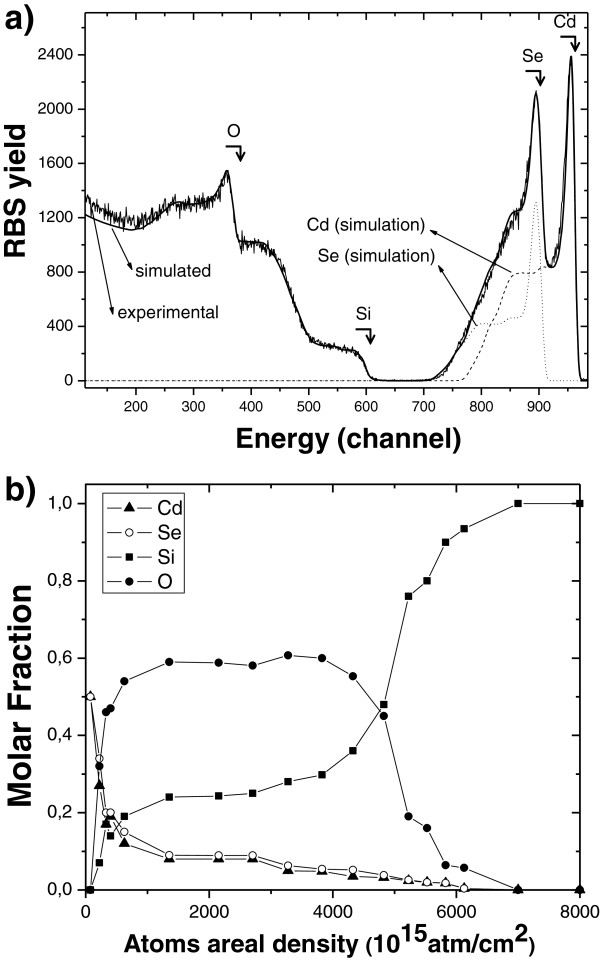
**RBS spectrum for a CdSe embedded PS sample and simulated composition profiles.** (**a**) RBS spectrum for a CdSe embedded PS sample using a 3.035-keV alpha-particle beam, indicating the regions corresponding with the different elements and (**b**) simulated composition profiles using SIMNRA software.

Figure 2 represents the diffractogram for a high porosity porous silicon sample in which CdSe was deposited using ICSS with exposure and purge time of 90 s for 8 cycles. The pattern was taken in grazing incidence geometry to avoid the strong reflections of the Si substrates. Peaks in the spectrum correspond to hexagonal CdSe. Figure 3 shows cross-sectional (Figure 3a) and top-view (Figure 3b) SEM micrographs of such a sample. As can be seen, the surface shows small grains of CdSe deposited on top of the porous surface. Some of the grains show a hexagonal geometry that confirms the previous XRD observation. For comparison, the top-view image of a pristine PS layer prepared using the same conditions of the CdSe embedded sample is shown in Figure 3c. The cross-section image shows a porous silicon film of around 1.4-μm thick. To investigate the CdSe semiconductor filling of the pores, RBS spectra were taken and analysed. Figure 4a shows the RBS spectrum for the sample shown in Figure 3. The spectrum was simulated using the SIMNRA software which allowed determining of its composition profile shown in Figure 4b.

According to these profiles, the sample can be divided in three regions. In region I (near surface region), the simulation of the spectrum indicates only a stoichiometric 1:1 CdSe film. This region represents only around 2% of the total thickness (in areal density of atoms units) of the films and can be related to the CdSe grains observed by SEM on the PS surface. In region II, the most part of the film (around 70% of the total thickness) shows a nearly constant composition of O, Si, Cd and Se. In this region, a stoichiometric Cd/Se proportion is observed. This supports the XRD measurements where no other phase different to CdSe is observed. In view of the CdSe stoichiometry, it can be assumed that oxygen present in the sample comes from SiO_2_ in the inner walls of the pores. From the compositional analysis, it can be concluded that CdSe has successfully infiltrated inside the PS layer down to the substrate in almost constant proportion, indicating a slow conformal CdSe growth inside the PS.

### ZnTe

**Figure 5:**
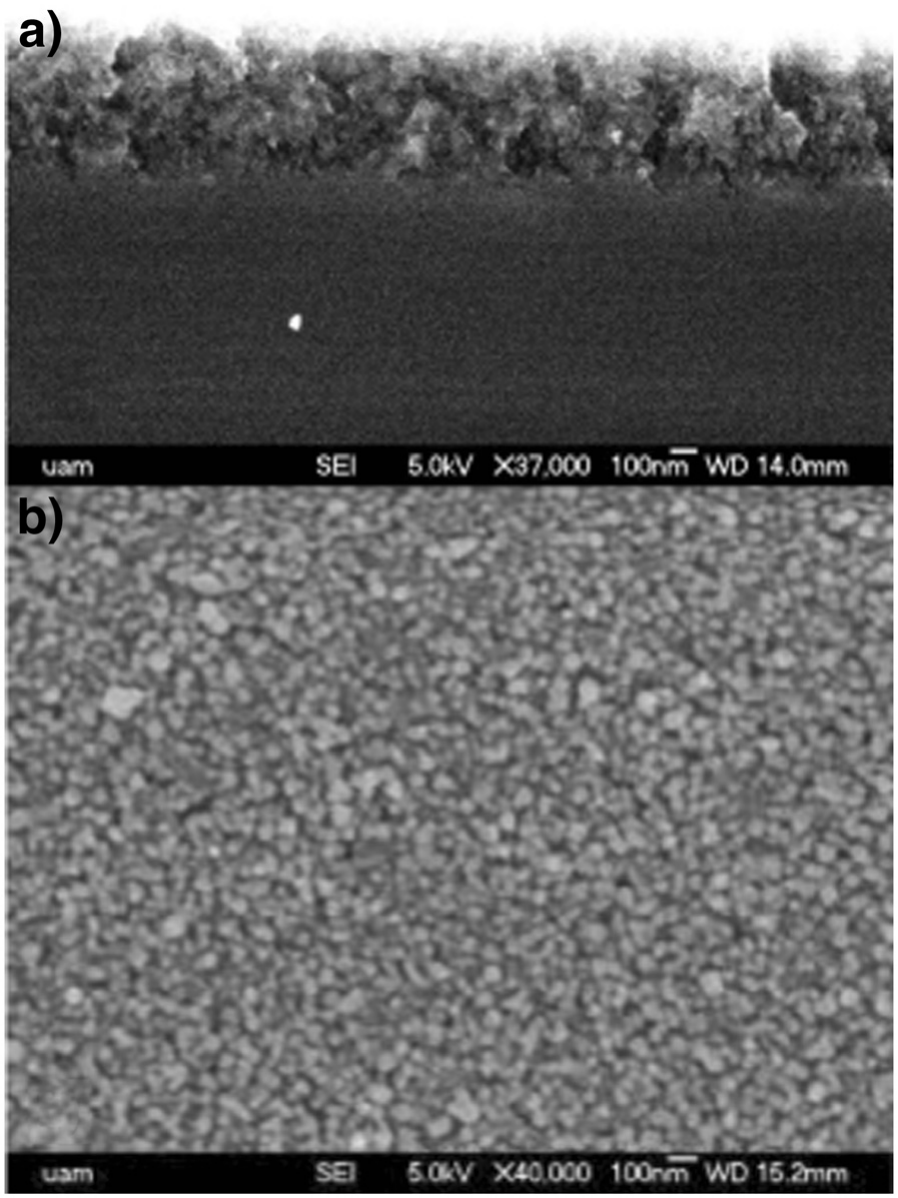
**SEM micrograph for a ZnTe embedded PS layer.** Cross section (**a**) and top view (**b**).

**Figure 6:**
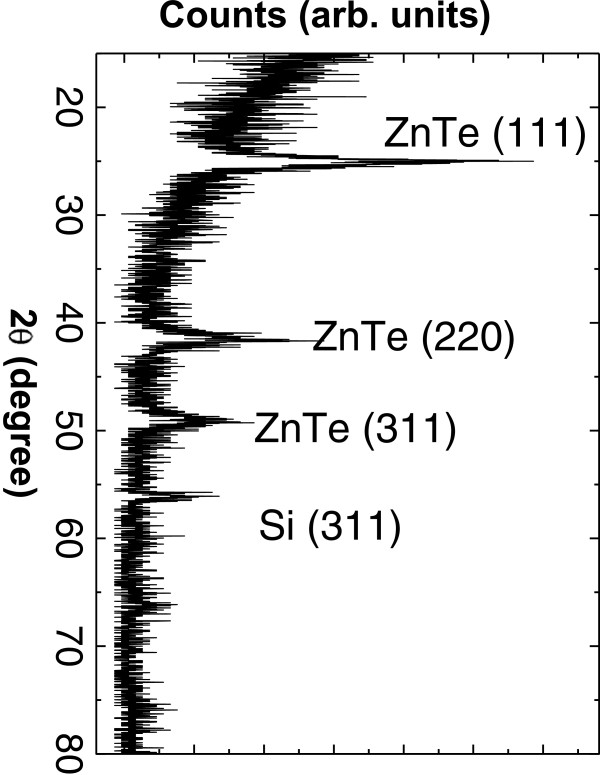
Grazing incidence XRD for ZnTe-embedded PS sample showing the zinc blende crystalline structure of ZnTe.

**Figure 7:**
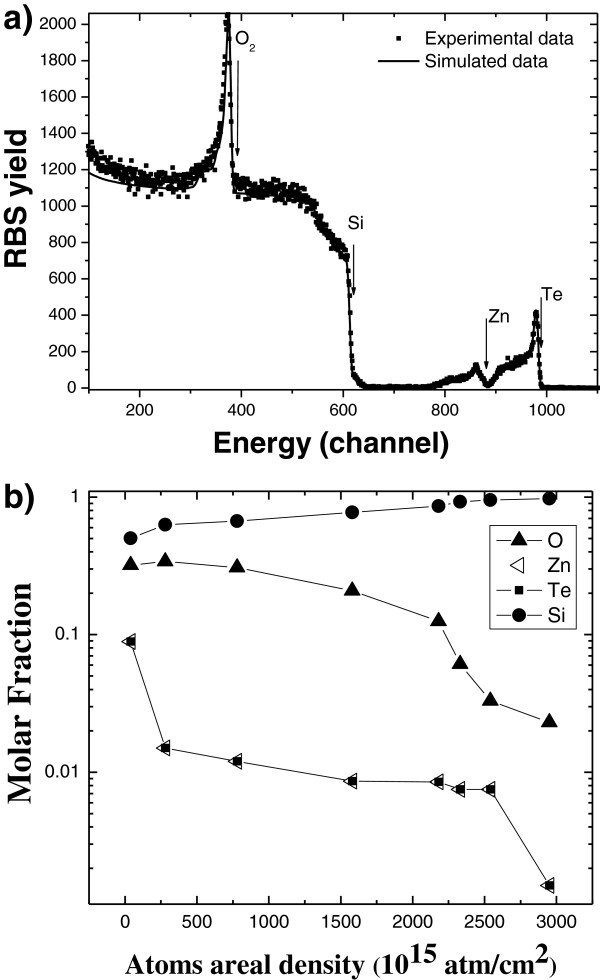
**RBS spectrum for a ZnTe-embedded PS sample and simulated composition profiles.** (**a**) RBS spectrum for a ZnTe-embedded PS sample using a 3.035-keV alpha-particle beam indicating the regions corresponding with the different elements and (**b**) simulated composition profiles using SIMNRA software.

Figure 5 shows SEM micrographs of the cross section (Figure 5a) and surface (Figure 5b) of a ZnTe-embedded PS low porosity sample with exposure and purge time of 90 s and 8 cycles. From the cross-section image, a thickness of 515 nm was measured. Several nanocrystals are observed in the surface of the sample in the range of around 10 to 100 nm. Figure 6 shows the grazing incidence X-ray diffractogram for the same sample. As can be seen in the figure, the more intense peaks corresponding to the zinc blende structure of ZnTe are observed. The peak at around 2*θ* = 56 corresponds to the background of the (311) diffraction of Si. In Figure 7a,b, the RBS spectrum and the composition profiles obtained by the spectrum simulation are shown. In this case, the PS layer is less evident in the RBS spectrum as compared with that of the high porosity sample (Figure 4) because in this case, there is a lower difference in Si content between the porous layer and the bulk substrate. The composition profile shows also three distinct regions: a higher ZnTe content near the surface, an almost constant composition region for the most part of the PS layer, with a stoichiometric ZnTe composition and a final region with decreasing Zn, Te and O content, which corresponds to the transition layer between PS and the bulk substrate. It can be observed that the amount of ZnTe in the PS layer is smaller in this case (a logarithmic scale was used to show the small ZnTe molar fraction in the profile) as expected from a lower porosity layer (i.e. with a lower void content). In this case, the simulation did not require a significant amount of porosity in the layer.

**Figure 8:**
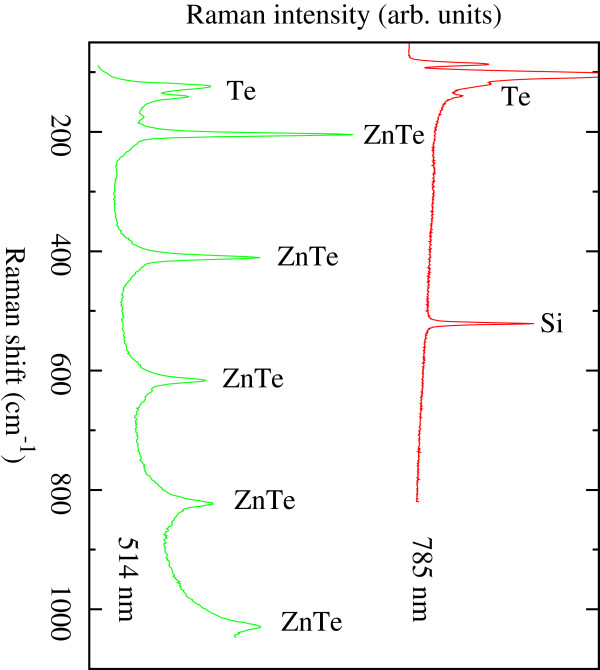
Raman spectra of samples measured for 785- and 514-nm laser wavelengths of ZnTe-embedded PS sample.

Raman spectra of the samples were measured for two laser wavelengths: 785 and 514 nm. A summary of the results are shown in Figure 8. The photon energy corresponding to a 785 nm wavelength is below the bandgap energy of bulk ZnTe. This, alongside the fact that the PS layer, is less than 2-μm thick allowing the probing beam to reach the substrate; hence, the spectrum is dominated by the crystalline silicon peak at 520 cm^−1^. The two Raman peaks at 127 and 141 cm^−1^ are related to crystalline Te precipitates [10] which are usually present in ZnTe and CdTe. Since these Te precipitates are strong Raman scatterers, they can reflect very tiny deviation of the stoichiometry (below the composition resolution of RBS). On the other hand, it has been observed that the laser would contribute to the precipitation or some Te in the surface [11]. No detectable peaks were obtained from ZnTe.

The laser wavelength of 514 nm (photon energy of 2.409 eV) is slightly above the ZnTe bandgap at room temperature. Therefore, most of the probing beam is absorbed; hence, the substrate Raman signal is barely visible in the spectrum. However, the Raman scattering from ZnTe is very strong. The longitudinal optical phonon mode of ZnTe appears at 204 cm^−1^. The peaks at multiples of this frequency correspond to the overtones of this fundamental phonon. The enhancement of the Raman signal for ZnTe and the appearance of the overtones may be attributed to a resonance effect indicating that the incident photon energy is very close to the semiconductor bandgap. In this case, the probing photon energy is 2,409 eV, which is 15 meV higher than the bulk ZnTe bandgap at room temperature. This enhancement of the ZnTe bandgap can be attributed to an electronic quantum confinement effect in ZnTe nanostructures. Assuming a spherical shape of the ZnTe clusters, a bandgap widening of 15 meV corresponds to cluster diameters of 13 nm.

## Conclusions

In this work, ZnTe and CdSe compounds have been grown in porous silicon by ICSS. XRD and RBS measurements revealed that stoichiometric polycrystalline ZnTe and CdSe are formed. Moreover, in-depth compositional analysis of the RBS data showed that the stoichiometry and content of the semiconductor compounds remain almost constant throughout the porous layer down to the substrate. A resonance in the Raman scattering of the ZnTe phase has been observed in porous silicon layers embedded with ZnTe at photon energies above the bulk ZnTe bandgap, indicating that the semiconductor is growing in nanostructured form inside the pores which induce electronic quantum confinement effects. Results presented in this paper suggest that isothermal close space sublimation is a promising technique for the conformal growth of II-VI semiconductor nanostructures in porous silicon.

## Competing interests

The authors declare that they have no competing interests.

## Authors’ contributions

VT-C prepared the porous silicon samples and participated in the design of the experiments in both the interpretation of the RBS spectra and in the writing of the paper. CdM performed the growth of ZnTe on the PS layers and collaborated in the simulation of RBS spectra. AC-F made the RBS measurements of all the samples and collaborated in the simulation of the spectra and in the writing of the paper. FA-R made Raman spectroscopy measurement as well as its interpretation and collaborated in the writing of the paper. OdM conceived and participated in the design of the experiments, and performed the growth of CdSe samples in PS layers, X-rays and RBS measurements and in the writing of the paper. All authors read and approved the final manuscript.
